# The effect of canagliflozin on hippocampal dendrite morphology in a model of Alzheimer’s disease induced by intracerebroventricular injection of streptozotocin

**DOI:** 10.1007/s00429-026-03142-4

**Published:** 2026-06-14

**Authors:** Sevdenur Yazi, Berna Ozen, Bahar Buldu, Ebru Yalcin, Osman Karakose, Ozan Cakmak, Selenay Somunkiran, Hasan R. Yananli, Umit S. Sehirli, Ozlem Kirazli

**Affiliations:** 1 School of Medicine, Department of Anatomy, Marmara University, Istanbul, Turkey; 2 Institute of Health Sciences, Marmara University, Istanbul, Turkey; 3 School of Medicine, Department of Medical Pharmacology, Mugla Sitki Kocman University, Mugla, Turkey; 4 School of Medicine, Marmara University, Istanbul, Turkey; 5 School of Medicine, Department of Medical Pharmacology, Marmara University, Istanbul, Turkey

**Keywords:** Alzheimer’s disease, Canagliflozin, Donepezil, Dendrite, Dendritic spine

## Abstract

Alzheimer’s disease (AD) and diabetes mellitus (DM) share common pathophysiological features. However, the effects of antidiabetic drugs on neurodegeneration are not completely known. Canagliflozin, a novel option for DM treatment, is a dual inhibitor of sodium glucose co-transporter type 2 (SGLT2) and acetylcholinesterase. The aim of this study is to examine the morphological features of dendrites and dendritic spines of pyramidal neurons in hippocampus of AD model treated with canagliflozin. The model of AD was obtained by intracerebroventricular injection of streptozotocin. Then, the rats were divided into 3 groups: vehicle, donepezil, and canagliflozin. The injections were i.c.v. administered for 7 days. Behavioral tests were performed to evaluate memory, anxiety, and motor functions. Brain tissues were processed by Golgi impregnation method. Pyramidal neurons in the CA1 region were examined using Neurolucida software. Dendritic branching, total dendrite length, dendritic spine density, and dendritic spine types were analyzed. Compared to the vehicle group, the donepezil group and the canagliflozin group exhibited significantly higher dendritic branches (*p* = 0.0273, *p* = 0.0195) and total dendrite length (*p* = 0.0171, *p* = 0.0360), respectively. The total dendritic spine density (*p* < 0.0001) and the mushroom-type dendritic spine density (*p* = 0.0001) were significantly low in the donepezil group compared to the vehicle group. However, canagliflozin did not induce any significant alterations in the dendritic spine density. Canagliflozin treatment was as effective as donepezil treatment on hippocampal dendrite morphology. This morphological framework, indicating dendritic plasticity and remodeling, serve to better understand the cellular effects of canagliflozin. Therefore, our study may contribute to the development of novel strategies for therapy of AD.

## Introduction

Alzheimer’s disease (AD), the most common type of dementia, is a slowly progressive neurodegenerative disorder. It causes the loss of memory, language, reasoning, spatial awareness, and mobility (Beata et al. 2023). It is characterized by neuritic plaques and neurofibrillary tangles as a result of the aggregation of amyloid-beta peptide (Aβ). The medial temporal lobe and neocortical structures are the most affected regions in the brain. Synaptic damage in these anatomical regions causes progressive loss of cognitive functions (Breijyeh and Karaman 2020).

Diabetes mellitus (DM), a chronic metabolic disease, is characterized by elevated blood glucose. The cause of the hyperglycemia is inadequate insulin production or lack of response to insulin. Type 1 diabetes (T1D) and type 2 diabetes (T2D) affect cognitive functions. Epidemiological studies have identified an increased AD risk in patients with DM. There is evidence that insulin also plays a role in AD. A connection between AD and insulin dysfunction is supported by biochemical analysis (El Khoury et al. 2014; Ke et al. 2009; Sun et al. 2020). On the other hand, there are numerous complicated links between AD and DM, including neuroinflammation and mitochondrial dysfunction. Therefore, AD is also considered as type 3 diabetes (Zhang et al. 2018).

Currently, AD treatments that just improve the symptoms are available. There are two classes of drugs approved: inhibitors to the cholinesterase enzyme and antagonists to N-methyl-D-aspartate (NMDA). Cholinesterase inhibitors donepezil, rivastigmine, and galantamine are used for symptomatic treatment. Donepezil, an acetylcholinesterase (AChE) inhibitor, is considered the leading drug for AD treatment (Breijyeh and Karaman 2020). Regarding common mechanisms (such as inflammation, insulin signaling alterations) between AD and DM, the effects of antidiabetic drugs on neurodegeneration are not completely known. Pharmacological agents used in DM treatment have been observed to have protective effects on cognitive impairments in AD (Nowell et al. 2023).

A novel option for DM treatment is sodium glucose co-transporter type 2 (SGLT2) inhibitors. The primary SGLT2 inhibitors are canagliflozin, dapagliflozin, and empagliflozin. The mechanism is independent of insulin. These oral antidiabetics decrease glucose reabsorption in the kidney and increase glucose excretion in the urine. Consequently, they cause a decrease in plasma glucose and glycated hemoglobin levels (Abdul-Ghani et al. 2011; Bailey 2011). Canagliflozin exhibits the greatest potential for inhibiting SGLT1 receptors among SGLT2 inhibitors. Moreover, canagliflozin has even been referred to as a dual inhibitor of SGLT2 and AChE (Pawlos et al. 2021).

Emerging therapeutic approaches for neurodegenerative diseases highlight restoring synaptic function and integrity. Therefore, both dendritic and axonal synapses are crucial for understanding neuronal network activity (Dejanovic et al. 2024; Wu et al. 2015). The central nervous system contains mostly axodendritic synapses. Axosomatic, axoaxonic, and dendrodendritic synapses are also present (Ovalle and Nahirney 2020). In this context, one of the factors that determines synaptic density and the dimension of the receptive area is the geometry of the dendritic arbor, which is specific to the neuronal subtype (Lanoue and Cooper 2019).

The primary receptives of synaptic inputs are dendritic spines, identified by neuroanatomist Cajal in 1888. Dendritic spines, which are quite heterogeneous in size and shape, are classified as thin type, mushroom type, stubby type, and branched type according to their morphological characteristics (Yuste 2023). Filopodia is an emerging kind of dendritic spine that extends from the dendritic shaft in search of a synaptic partner (Stratton and Khanna 2020). During synaptogenesis, filopodia is produced in large numbers and could be precursors of numerous dendritic spines (Yuste 2023). Alterations in the sizes, shapes, and densities of these structures are related to the strength of synaptic connections (Hotulainen and Hoogenraad 2010).

Dysregulation in structural and functional plasticity mechanisms of dendrites and dendritic spines is associated with neurodegenerative and neuropsychiatric diseases as well as epilepsy (Lanoue and Cooper 2019; Stein and Zito 2019; Yazi et al. 2024). Synaptic loss mechanisms (accumulation of Aβ and tau at the synaptic sites, defects in axonal transport, mitochondrial damage, and oxidative stress) in AD lead to a loss of dendritic spines (Breijyeh and Karaman 2020). There are studies in the literature that found a decrease in dendritic complexity and number of dendritic spines in AD (Cochran et al. 2014; Stein and Zito 2019). Studies with the AD model induced by streptozotocin (STZ) revealed that the STZ group had a significantly lower total number of dendritic spines (particularly mushroom type) of the CA1 region compared to the control group (Gupta et al. 2018; Xu et al. 2014, 2018).

Since AD and DM share common pathophysiological features, it is inevitable to evaluate the therapeutic potential of SGLT2 inhibitors in AD (Katsenos et al. 2022). There are studies examining the effects of canagliflozin, dapagliflozin, and empagliflozin on cognitive functions (Apaijai et al. 2025; Arafa et al. 2017; Hierro-Bujalance et al. 2020; Ibrahim et al. 2022). However, no studies have demonstrated the effect of canagliflozin on dendrite morphology, which is associated with synaptic function and plasticity, in AD. The aim of this study is to examine the morphological features of dendrites and dendritic spines of pyramidal neurons in hippocampus of AD model treated with canagliflozin and donepezil.

## Materials and methods

### Animals

This study protocol is approved by the Marmara University Local Ethics Committee for Animal Experiments (Protocol Code: 54.2022mar). The experimental animals used in this study were provided by the Marmara University Experimental Animals Research and Implementation Center.

A total of 18 adult male *Wistar* rats (290 ± 10 g) were used in this study. The animals were kept in a room with a controlled temperature (21 ± 2 °C) and relative humidity (45–65%) under a 12-hour light/dark cycle. Animals had unlimited access to standard rat chow and water. The animals were randomly divided into 3 groups. The experimental groups in detail were as follows:

1. STZ injected + Vehicle (Dimethyl sulfoxide) (*n* = 6).
2. STZ injected + Donepezil (*n* = 6).
3. STZ injected + Canagliflozin (*n* = 6).

**Fig. 1:**
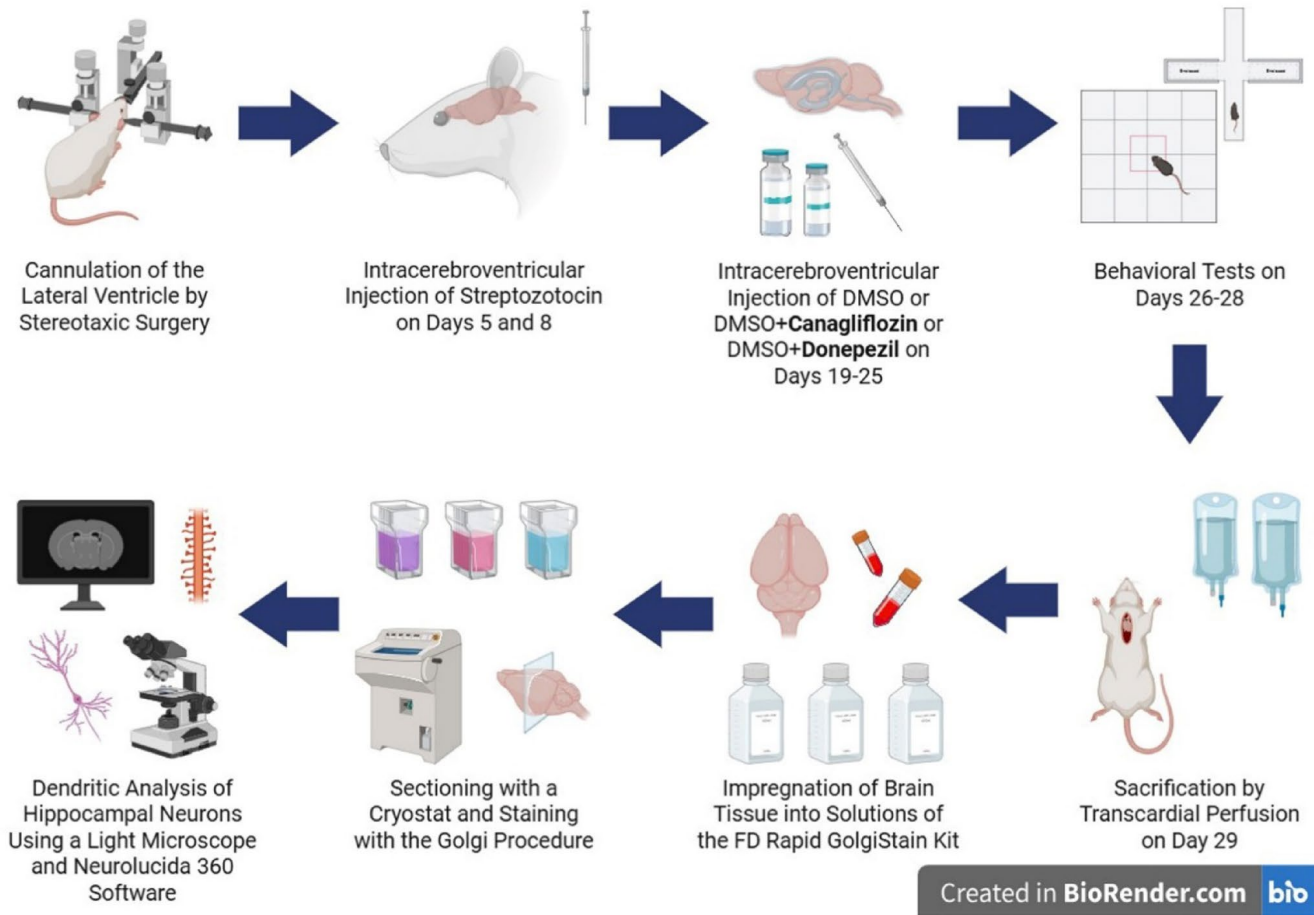
Methodology of the experiment

The experimental method is summarized in Fig. 1.

### Alzheimer’s disease model

The model of AD was obtained by intracerebroventricular injection of STZ (Salkovic-Petrisic and Hoyer 2007). For this purpose, a unilateral cannula was implanted into the lateral ventricle by stereotaxic surgery on the first day of the experiment. Following the deep anesthesia with ketamine (100 mg/kg i.p.) and xylazine (10 mg/kg i.p.), the rat’s skull was placed in the stereotaxic instrument (World Precision Instruments) through the external acoustic meatus. The scalp was incised sagittally, and the periosteum was stripped. The cannula insertion site and 4 points located ~ 1 cm distal to it were drilled in accordance with the coordinates of the stereotaxic atlas (Paxinos and Watson 2006). A catheter was inserted into the right lateral ventricle (1.0 mm caudal from the bregma, 1.5 mm lateral from the sagittal suture, and 3.5 mm inward from the skull surface). Screws were inserted into the other 4 points to enhance the fixation of the cannula. Then, the cannula and its surroundings were fixed using acrylic. A 5-day waiting period was allowed before initiating STZ injection to enable natural recovery of the rats after stereotaxic surgery.

On the 5th and 8th days, the rats were induced by intracerebroventricular injection of STZ (3 mg/kg, Sigma S0130) dissolved in citrate buffer solution. 14 days were allowed for the formation of the model.

### Experimental groups

On the 19th -25th days, the experimental groups were injected with vehicle (20% DMSO i.c.v., Merck 1.16743.1000), donepezil (dissolved in 20% DMSO, 0.1 mg/kg i.c.v., Cayman 13245) (positive control), and canagliflozin (dissolved in 20% DMSO, 3 µM i.c.v., Cayman 11575), respectively. The injections were administered for 7 days via the guide cannula using a Hamilton microliter syringe, a polyethylene tube, and an internal cannula.

### Behavioral tests

Behavioral tests were conducted to both confirm the AD model and evaluate the experimental groups. On the 26th day, the elevated plus maze and the locomotor activity test were used to evaluate anxiety behavior and motor functions, respectively. The novel object recognition test (on the 26th -28th days) and the passive avoidance test (on the 26th -29th days) were used to evaluate learning and memory. Behavioral tests were performed between 8 am and 12 pm. Before the tests began, the animals were accustomed to the laboratory for 30 min.

#### Elevated plus maze

The maze has four arms (two open and two enclosed) in a plus shape and is elevated approximately 50 cm. The rats were placed facing an open arm at the junction of the four arms of the maze. Entries and durations in each arm were recorded by an observer for 5 min. Between tests, the maze was cleaned with 70% ethanol and allowed to dry. An increase in open arm activity reflects anti-anxiety behavior (Walf and Frye 2007).

#### Locomotor activity test

The locomotor activity test allows a comprehensive examination of the locomotor activity levels of experimental animals. Activity meter (Commat Ltd., ACT 508), which is a 40 × 40 × 40 cm cage, records the locomotor activity parameters such as ambulatory, horizontal and vertical activities of animals.

The rats were placed in the cage, and all movements were recorded for 5 min. The alterations of location and position were recorded in the cage’s software via horizontal and vertical motion sensors inside the cage. Between tests, the cage was cleaned with 70% ethanol and allowed to dry.

#### Novel object recognition test

The novel object recognition test performed in a 40 × 40 × 40 cm cage consists of 3 phases: habituation, training, and testing. On the 26th day, the rats were accustomed to the environment for 10 min in the cage devoid of any objects (habituation). On the 27th day, two identical objects (A-A) were located symmetrically on the cage floor. Then, the rats were placed in the cage, and the examination times of the two objects were recorded for 5 min (training). The examination of the objects was defined as a max distance of 1 cm between the rat’s nose and the object and/or the rat touching the object with its nose. 15 min after the training phase, two different objects (A-B) were located symmetrically on the cage floor. Then, the rats were placed back in the cage, and the examination times of the two objects were recorded for 5 min (testing). This stage evaluated short-term memory. On the 28th day, two different objects (A-C) were located symmetrically on the cage floor. Then, the rats were placed in the cage, and the examination times of the two objects were recorded for 5 min (testing). This stage evaluated long-term memory. Between tests, the cage was cleaned with 70% ethanol and allowed to dry.

The ratio index was calculated by dividing the examination time of the new object by the total examination time of the objects and multiplying by 100. A higher ratio index indicates better visual memory retention.

#### Passive avoidance test

The passive avoidance test consists of two compartments: a bright chamber (safe area) and a dark chamber (unsafe area). The floor of the dark chamber is equipped with an electric grill.

On the 26th day, the rats were placed in the bright chamber and allowed to move instinctively to the dark chamber. After the rat entered the dark chamber, the guillotine-like sliding door between the two compartments was closed. In this chamber, an electrical shock (0.3–0.6 mA) was applied to the rat’s feet for 5 s. On the 29th day, the rats were placed back in the bright chamber, and the times to enter the dark chamber were recorded. Rats with normal memory are expected to avoid entering the dark chamber associated with the electrical shock for 5 min (cut-off). A shorter time for rats to enter this chamber indicates impaired memory.

### Tissue preparation

Tissue preparation was performed according to the instructions of the FD Rapid GolgiStain Kit (FD NeuroTechnologies, Inc.). The FD Rapid GolgiStain Kit contains Solutions A, B, C, D, and E.

Impregnation solution (Solution A + B) was prepared by mixing equal volumes of Solutions A and B at least 24 h prior to sacrification. The impregnation solution was stored at room temperature in the dark before use. The rats were deeply anesthetized with ketamine (100 mg/kg) and xylazine (10 mg/kg) intraperitoneally. Then, the rats were transcardially perfused with 4% paraformaldehyde in 0.1 M phosphate buffer (pH 7.4) after initially receiving 0.9% saline. Following perfusion, the brains were removed and divided into three blocks coronally with a sharp blade. The tissues were immersed in the impregnation solution (Solution A + B) and stored at room temperature for 3 weeks in the dark. Then, the tissues were transferred into Solution C and stored at room temperature for 1 week in the dark. 200 μm-thick coronal sections were obtained from all three blocks using a cryostat (Leica CM1950) at -20 °C to -23 °C. The sections were mounted on positive-charged microscope slides using Solution C and allowed to dry naturally at room temperature in the dark.

### Staining procedure

The Golgi staining procedure was performed, taking care not to dry out the sections between any steps. Firstly, staining solution (Solution D + E) was prepared freshly. The staining solution consists of 1 part Solution D, 1 part Solution E, and 2 parts double distilled water. Sections were rinsed in double distilled water 2 times for 4 min each time and placed in the staining solution (Solution D + E) for 10 min. Sections were rinsed again in double distilled water 2 times for 4 min each time. Then, sections were dehydrated in sequential rinses of 50%, 75%, and 95% ethanol, 4 min each and dehydrated in 100% ethanol 4 times for 4 min each. Sections were cleaned in xylene 3 times for 4 min each. Finally, sections were coverslipped with Permount^®^ mounting medium. Golgi-stained sections were stored at room temperature and protected from light.

### Neurolucida analysis

Sections stained with Golgi were examined using an Olympus BX51 microscope and a QImaging Retiga-2000R camera. Three-dimensional images were analyzed using Neurolucida 360 software (MBF Bioscience). Morphological analysis of 6 pyramidal neurons in the CA1 region of the hippocampus was performed in each rat brain according to the rat brain atlas as a reference. The CA1 region was analyzed between bregma AP -3.12 mm and − 4.20 mm (Paxinos and Watson 2006).

**Fig. 2:**
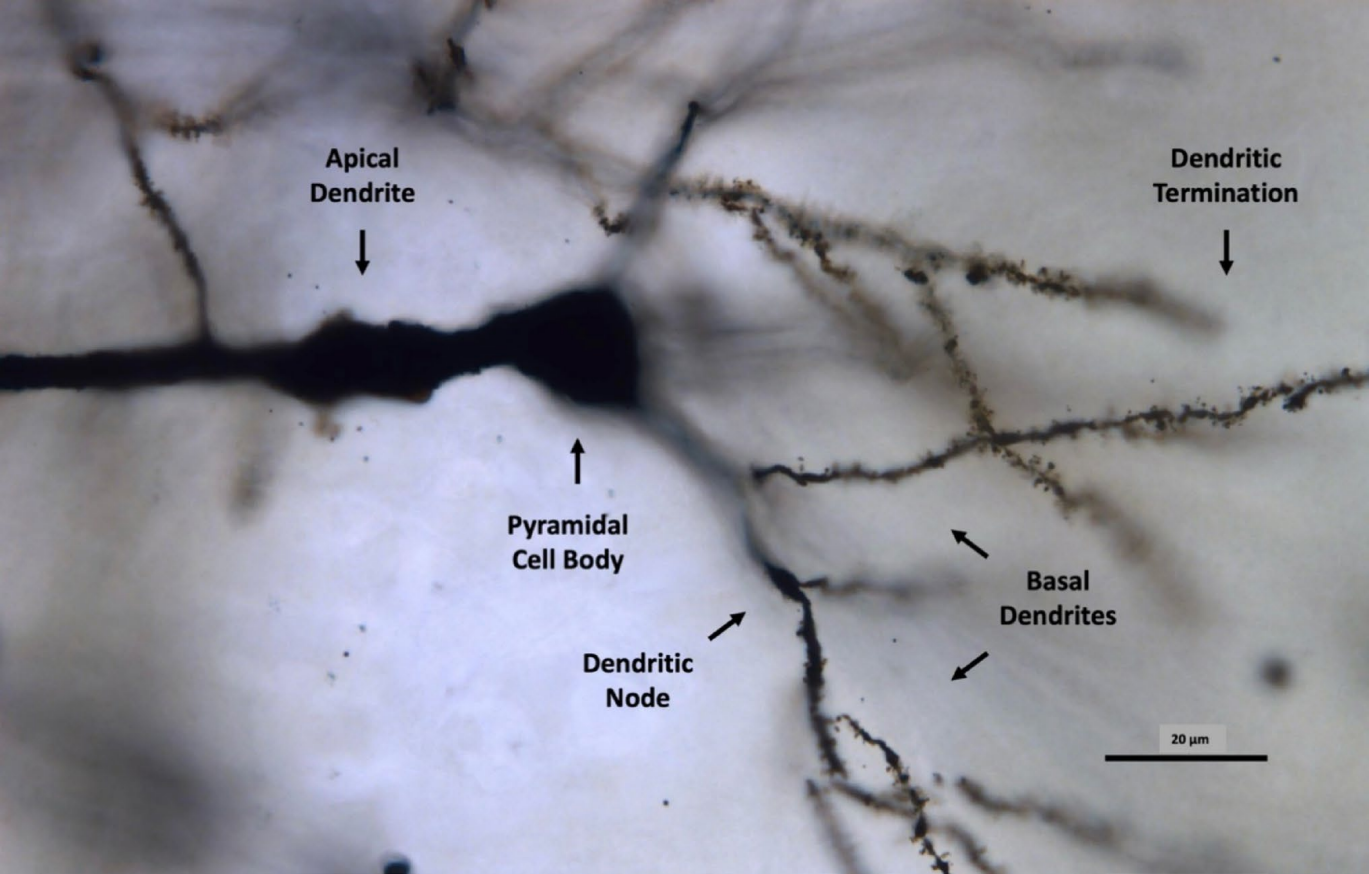
Dendrites of a pyramidal neuron of the AD model induced by STZ. The image shows the soma, apical dendrite, basal dendrites, and also dendritic spines on dendritic branches (x60 magnification). The bifurcation or trifurcation points of the dendrites were called dendritic nodes. The end points of the terminal dendrites were called dendritic terminations

Soma and dendrites were identified using three-dimensional images taken with a 60x objective. As a result of this reconstruction, data regarding the number of dendrite nodes, the number of dendrite terminations, the number of dendrite segments (dendritic branching), and the total dendrite length (µm) were obtained (Fig. 2).

**Fig. 3:**
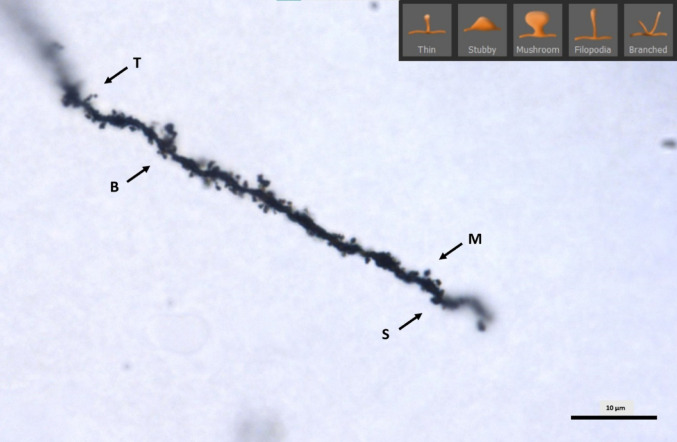
Dendritic spines of a pyramidal neuron of the AD model induced by STZ. The image shows thin-type (T), stubby-type (S), mushroom-type (M), branched-type (B), and filopodia (x100 magnification). The dendritic spine types in the right corner are taken from the software

Dendritic spines on a terminal branch of basal dendrites (30 μm length) were identified using three-dimensional images taken with a 100x objective. Dendritic spines were classified as thin type, stubby type, mushroom type, branched type, and filopodia. Classification was made according to the difference between the head part and the neck part of the dendritic spine. Thin-type dendritic spines were defined as those in which the diameter of the neck was less than total spine length and the diameter of the head was greater than that of the neck. Dendritic spines lacking a neck part were defined as stubby type. Dendritic spines with the diameter of the head part greater than the diameter of the neck part were defined as mushroom type. Filopodia was defined as a dendritic structure which is longer (3–10 μm) than the other types. Dendritic spines with one neck part and two head parts were defined as branched type (Yuste 2023). As a result of this reconstruction, data regarding the total density of the dendritic spine (1/µm) and the individual density of the dendritic spine types (1/µm) were obtained (Fig. 3).

### Statistical analysis

All data were expressed as mean ± standard error of the mean (SEM) and analyzed using GraphPad Prism 8.3.0 program. Morphological parameters in the hippocampus of the experimental groups were compared with one-way ANOVA followed by post-hoc Tukey test. Statistical significance was considered at *p* < 0.05.

## Results

Our results revealed significant differences in the morphology of dendrites and dendritic spines, the structural and functional components of neurons, between the experimental groups. This suggests that canagliflozin and donepezil cause significant synaptic reorganization in the examined brain regions.

### Morphology of dendrites in hippocampus

**Fig. 4:**
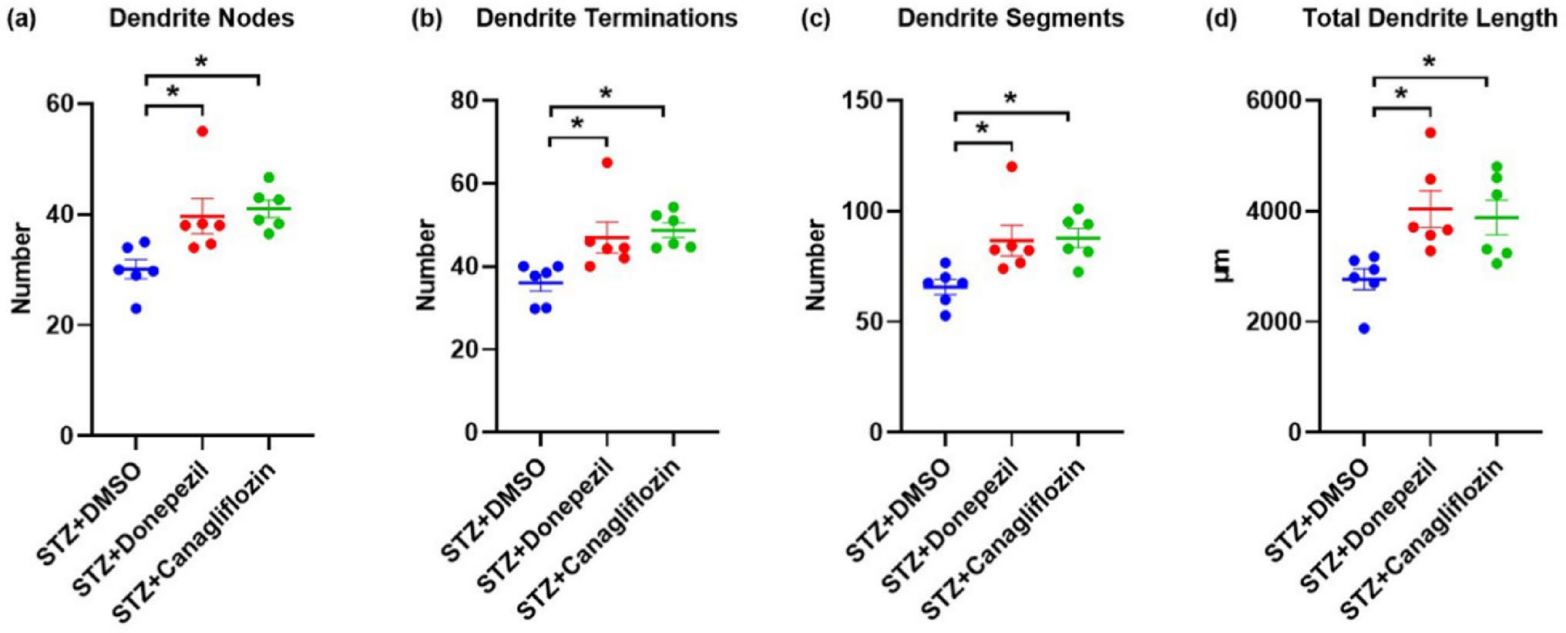
Comparison of dendrite parameters between experimental groups. Comparison of **a** the number of dendrite nodes, **b** the number of dendrite terminations, **c** the number of dendrite segments, and **d** the total dendrite length in the CA1 region of the hippocampus between the STZ+DMSO group (vehicle), STZ+Donepezil group (positive-control) and STZ+Canagliflozin group (treatment). Data are shown as mean ± SEM. One-way ANOVA (post-hoc Tukey test), * *p* < 0.05, ** *p* < 0.01, *** *p* < 0.001, **** *p* < 0.0001

The dendrite morphology of the pyramidal neurons in the CA1 region of the hippocampus of the treatment groups showed statistically significant differences (Figs. 4 and 6).

The canagliflozin group (41.03 ± 1.530) had significantly higher number of dendrite nodes (*p* = 0.0102) compared to the DMSO group (30.13 ± 1.741). The canagliflozin group (48.72 ± 1.774) had significantly higher number of dendrite terminations (*p* = 0.0100) compared to the DMSO group (36.01 ± 1.964). The canagliflozin group (87.86 ± 4.318) had significantly higher number of dendrite segments (dendritic branching) (*p* = 0.0195) compared to the DMSO group (65.74 ± 3.387). Furthermore, the total dendrite length (*p* = 0.0360) was higher in the canagliflozin group (3884 ± 314.1) compared to the DMSO group (2769 ± 192.1).

Similarly, the donepezil group (39.67 ± 3.160) had significantly higher number of dendrite nodes (*p* = 0.0238) compared to the DMSO group. The donepezil group (46.97 ± 3.707) had significantly higher number of dendrite terminations (*p* = 0.0255) compared to the DMSO group. The donepezil group (86.64 ± 6.863) had significantly higher number of dendrite segments (dendritic branching) (*p* = 0.0273) compared to the DMSO group. Moreover, the total dendrite length (*p* = 0.0171) was higher in the donepezil group (4037 ± 328.5) compared to the DMSO group. However, there was no statistically significant difference in the dendrite morphology between the canagliflozin group and the donepezil group (Fig. 4).

### Morphology of dendritic spines in hippocampus

**Fig. 5:**
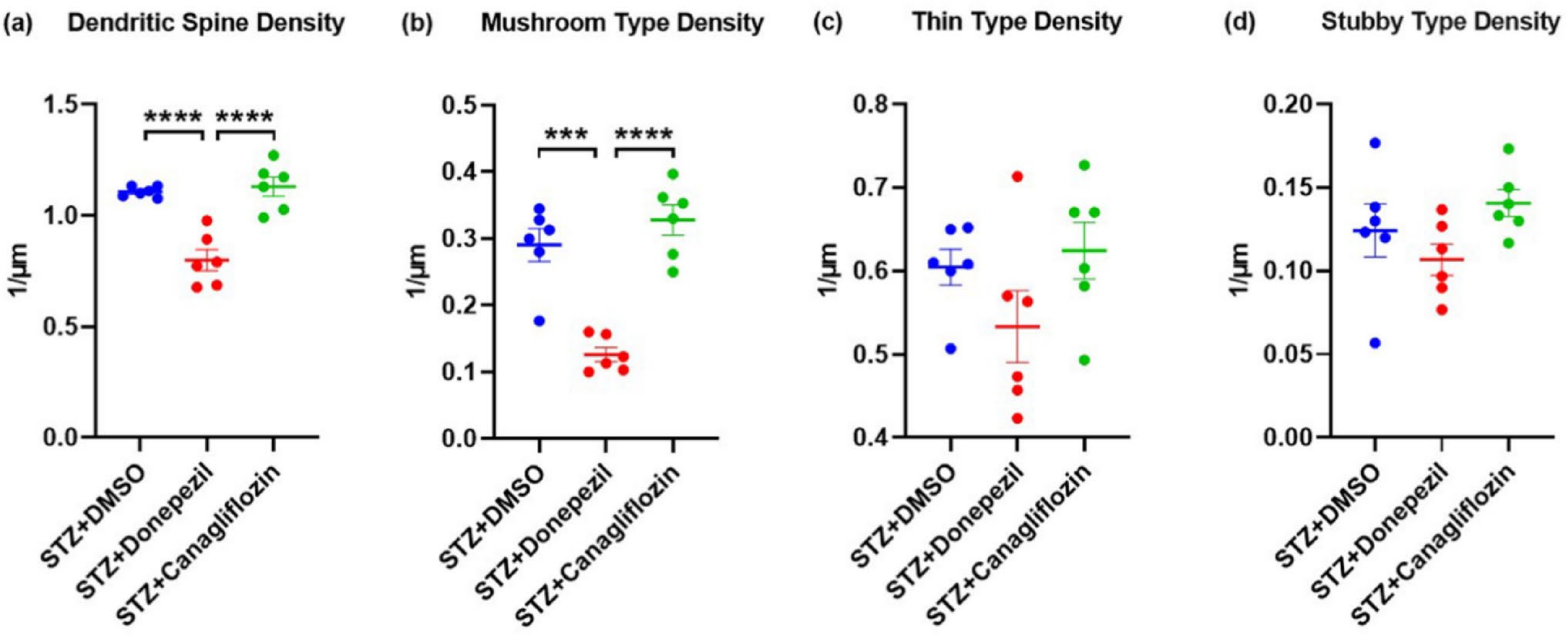
Comparison of dendritic spine parameters between experimental groups. Comparison of **a** the dendritic spine density, **b** the mushroom-type dendritic spine density, **c** the thin-type dendritic spine density, and **d** stubby-type dendritic spine density in the CA1 region of the hippocampus between the STZ+DMSO group (vehicle), STZ+Donepezil group (positive-control) and STZ+Canagliflozin group (treatment). Data are shown as mean ± SEM. One-way ANOVA (post-hoc Tukey test), * *p* < 0.05, ** *p* < 0.01, *** *p* < 0.001, **** *p* < 0.0001

**Fig. 6:**
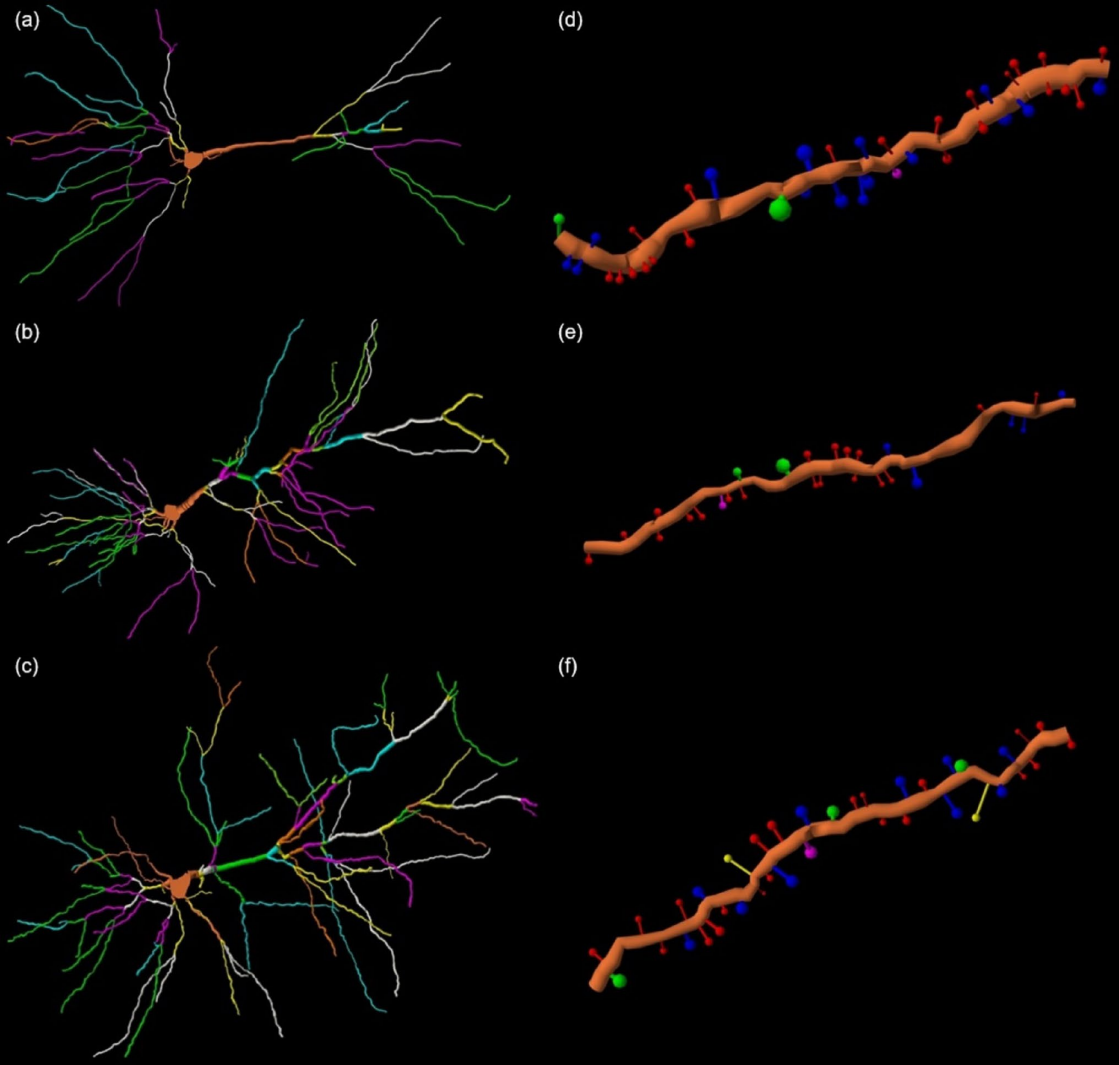
Neurolucida images reconstructing dendrites and dendritic spines of the experimental groups.Neurolucida 360 software colored dendrites according to their segmentation. Primary, secondary and tertiary dendrites represented by different colors. 3D reconstruction image of the dendrites of a pyramidal neuron in the CA1 region of the hippocampus of **a** DMSO group, **b** donepezil group, and **c** canagliflozin group. 3D reconstruction image of the dendritic spines of a pyramidal neuron in the CA1 region of the hippocampus of **d** DMSO group, **e** donepezil group, and **f** canagliflozin group. Dendritic spines: thin type (red), stubby type (green), mushroom type (blue), branched type (pink), and filopodia (yellow)

The dendritic spine morphology of the pyramidal neurons in the CA1 region of the hippocampus of the treatment groups showed statistically significant differences (Figs. 5 and 6).

There was no statistically significant difference in the total density of dendritic spines between the DMSO group and the canagliflozin group. However, the donepezil group (0.7994 ± 0.04788) had significantly lower total density of dendritic spines (*p* < 0.0001) compared to the DMSO group (1.107 ± 0.009510). When dendritic spine types were evaluated separately, mushroom-type dendritic spines (*p* = 0.0001) were lower in the donepezil group (0.1261 ± 0.01073) compared to the DMSO group (0.2906 ± 0.02455). There was no statistically significant difference in the individual density of the other types between the experimental groups (Fig. 5).

.

### Behavioral tests

**Fig. 7:**
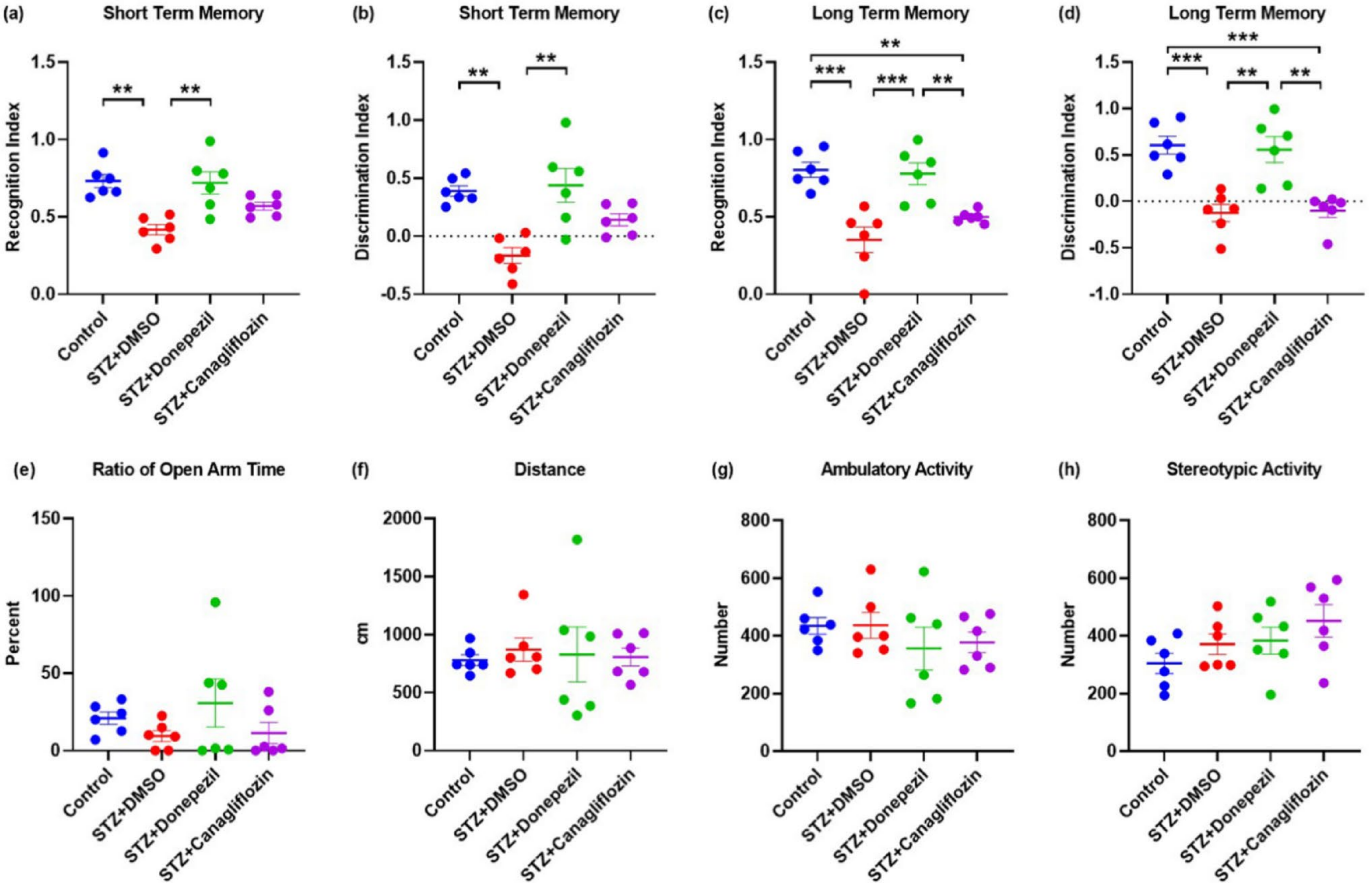
Comparison of behavioral parameters between experimental groups. Comparison of **a** the recognition index of the short-term memory (NORT), **b** the discrimination index of the short-term memory (NORT), **c** the recognition index of the long-term memory (NORT), **d** the discrimination index of the long-term memory (NORT), **e** the ratio of open arm time (EPM), **f** the distance (LAT), **g** the ambulatory activity (LAT), and **h** the stereotypic activity (LAT) between the control group (naive), STZ+DMSO group (vehicle), STZ+Donepezil group (positive-control) and STZ+Canagliflozin group (treatment). The control group refers to rats without the AD. Data are shown as mean ± SEM. One-way ANOVA (post-hoc Tukey test), * *p* < 0.05, ** *p* < 0.01, *** *p* < 0.001, **** *p* < 0.0001. NORT: Novel object recognition test, EPM: Elevated plus maze, LAT: Locomotor activity test

The short-term memory of the novel object recognition test showed statistically significant differences. The recognition index (*p* = 0.0011) and the discrimination index (*p* = 0.0023) were higher in the control group compared to the DMSO group. Similarly, the recognition index (*p* = 0.0051) and the discrimination index (*p* = 0.0029) were higher in the donepezil group compared to the DMSO group (Fig. 7).

The long-term memory of the novel object recognition test showed statistically significant differences. The recognition index (*p* = 0.0003) and the discrimination index (*p* = 0.0008) were higher in the control group compared to the DMSO group. Similarly, the recognition index (*p* = 0.0010) and the discrimination index (*p* = 0.0030) were higher in the donepezil group compared to the DMSO group. Furthermore, the canagliflozin group had significantly lower recognition index (*p* = 0.0013, *p* = 0.0043) and discrimination index (*p* = 0.0003, *p* = 0.0011) compared to the control group and the donepezil group, respectively (Fig. 7).

There was no statistically significant difference in the passive avoidance test, the elevated plus maze, and the locomotor activity test between any groups (Fig. 7).

## Discussion

In the present study, the morphological features of dendrites and dendritic spines of pyramidal neurons in the hippocampus of AD model treated with canagliflozin and donepezil were quantitatively analyzed.

The number of dendrite nodes, the number of dendrite terminations, the number of dendrite segments, and the total dendrite length in the CA1 region of the hippocampus were significantly higher in the donepezil group compared to the DMSO group. Similarly, the number of dendrite nodes, the number of dendrite terminations, the number of dendrite segments, and the total dendrite length in the CA1 region of the hippocampus were significantly higher in the canagliflozin group compared to the DMSO group. In brief, both treatments increased dendritic branching and total dendrite length in the AD model.

Interactions between cholinergic and glutamatergic systems can modulate forms of synaptic plasticity in the hippocampus (de Sevilla et al. 2008). The hippocampus is an essential region of the brain for learning and memory (Kostrzewska et al. 2025). Pyramidal neurons in the hippocampus are one of the principal excitatory cells. They use glutamate as the major neurotransmitter. Glutamatergic synapses are frequently located on dendritic spines, but they can be found directly on the dendritic shaft (Bucher et al. 2020). Glutamate, which has a critical role in excitability, is a significant regulator of dendrite growth. For instance, NMDA receptor activation increases dendrite number and total dendrite length (Kuo et al. 2010; Stratton and Khanna 2020). Based on this information, increased dendritic arborization and total dendrite length in the treatment groups can be associated with glutamatergic activity.

In this study, the morphology of dendritic spines as well as dendrites is presented in detail in order to get an idea about synaptic transmission in the treatment groups. Morphological features of actin-rich dendritic spines are associated with synaptic function and plasticity. Alterations in the morphology of these postsynaptic structures play a role in the establishment and reorganization of connectivity within neuronal circuits (Penzes et al. 2011). The balance between elimination, maturation, and plasticity of dendritic spines is extremely important for proper brain function (Pchitskaya and Bezprozvanny 2020).

The density of dendritic spines in the CA1 region of the hippocampus was significantly lower in the donepezil group compared to the DMSO group. However, there was no statistically significant difference in the density of dendritic spines in the CA1 region of the hippocampus between the canagliflozin group and the DMSO group. The lack of increase in dendritic spine density for both treatments may indicate that synaptic efficacy occur relatively in the dendrites of the neurons.

Mulholland et al. investigated the effects of donepezil in adult rats exposed to adolescent intermittent ethanol. Researchers found that this exposure reduced dendritic spine density, and donepezil treatment (by intragastric gavage) reversed the dendritic spine adaptations in hippocampus (Mulholland et al. 2018). Jian et al. established a bilateral common carotid artery occlusion model to simulate the pathology of vascular dementia. Unlike our study, donepezil dissolved in sodium carboxymethyl cellulose was orally administered. Donepezil treatment significantly restored the dendritic spines density in cortex and hippocampus (Jian et al. 2020). Ongnok et al. investigated the effects of donepezil on brain responses in cases of cardiac ischemia/reperfusion injury. In contrast to our study, donepezil dissolved in normal saline solution was intravenously administered through the femoral vein. Donepezil treatment significantly increased the number of dendritic spines in the CA1 region (Ongnok et al. 2021). In our study, donepezil dissolved in DMSO was i.c.v. administered, so it is possible that donepezil affected dendritic spine density differently from these studies. The results of our study were compatible with other studies. De Bartolo et al. studied animals subjected to cholinergic lesion. Donepezil was intraperitoneally administered, and layer III of parietal cortex were analyzed. As a result of the study, lower dendritic spine density in both apical and basal dendrites was observed in the donepezil group (De Bartolo et al. 2009). Likewise, Belyaev et al. studied transgenic APP/PS1 mice with a model of AD. Donepezil was intraperitoneally adminis tered, and entorhinal cortex were analyzed. There was no increase in dendritic spine density in the donepezil group (Belyaev et al. 2025). The cognitive-enhancing effect of donepezil may not be directly due to an increase in dendritic spine density, but rather through suppressing neuroinflammation (microglia/astrocyte activation), breaking down brain insulin resistance, reducing oxidative stress, or improving mitochondrial function (Kim et al. 2021). The ICV-STZ model sometimes causes hyperexcitability in neurons. The decrease in dendritic spine density may actually be an adaptive and protective response of the cell to protect itself from glutamate toxicity. Cognition may have improved because hyperexcitation has decreased (Shen et al. 2010).

Dendritic spines are classified as thin type, mushroom type, stubby type, branched type, and filopodia according to their morphological characteristics (Yuste 2023). In some studies, thin type and filopodia are qualified as small dendritic spines, and mushroom type and stubby type are qualified as large dendritic spines. Rapidly changing small dendritic spines can become large dendritic spines or vanish entirely (Kasai et al. 2003).

Only mushroom-type dendritic spines were lower in the donepezil group compared to the DMSO group. However, there was no significant difference in the individual density of the other types in the CA1 region of the hippocampus between the experimental groups.

Shapes of dendritic spines have been related to specific components of cognitive processes, such as learning or memory (Burgos et al. 2012). In the only study examining the effect of donepezil on dendritic spine types, it was observed that donepezil had no effect on the types of dendritic spines in the AD mice model (Belyaev et al. 2025).

The novel object recognition test assesses hippocampal-linked recognition memory in experimental animals. It is based on the tendency for animals to spend more time investigating a novel object than a familiar one (Wu et al. 2020). The short-term memory and the long-term memory were lower in the DMSO group (AD) compared to the control group (non-AD). These results between the DMSO group and the control group confirm the formation of the AD model induced by STZ. Donepezil had beneficial effects on both short-term and long-term memories. Canagliflozin was insufficient to ameliorate short-term and long-term memories.

The passive avoidance test assesses conditional avoidance learning in experimental animals. The difference from the novel object recognition test is that the amygdala plays a crucial role for aversive learning (McGaugh 2004). As a result of this test, there was no significant difference between the experimental groups.

The elevated plus maze evaluates anxiety levels of experimental animals. An increase in open arm activity reflects anti-anxiety behavior. As a result of this test, there was no significant difference in ratio of open arm time between the experimental groups. This result reveals that the AD model induced by STZ does not produce an anxiety phenotype and that both treatments have no anxiolytic/anxiogenic effects.

The locomotor activity test is one of the open field tests. It allows a comprehensive examination of the locomotor activity levels of experimental animals. As a result of this test, there was no significant difference in distance, ambulatory activity, and stereotypic activity between the experimental groups. These results reveal that general motor functions are preserved in the AD model induced by STZ and that both treatments have no effect on motor functions.

Donepezil is used to improve AD related cognitive impairment (Pepeu and Giovannini 2009). There are studies in the literature that donepezil could rescue the cognitive function in AD model. Cutuli et al. show that donepezil pre-treatment exerts beneficial effects on memory deficits induced by cholinergic depletion. Similar to our study, donepezil (i.p.) did not affect anxiety related behavior in the elevated plus maze (Cutuli et al. 2013). Nagakura et al. investigated the effect of orally administered donepezil on working memory and spatial memory in a mouse model of AD with accelerated Aβ production. Researchers conclude that acute treatment with donepezil at clinically relevant doses ameliorated memory deficits (Nagakura et al. 2013). Kim et al. reported that orally administered donepezil have a protective effect against memory deficits induced by Aβ oligomer (Kim et al. 2014). Other studies observed that orally administered donepezil improved the cognitive function in amyloid precursor protein/presenilin-1 transgenic mice (Easton et al. 2013; Guo et al. 2015; Zhang et al. 2012).

SGLT2 inhibitors are nominated as a promising class of drugs against AD symptoms. Sa-Nguanmoo et al. show that dapagliflozin enhanced hippocampal synaptic plasticity (Sa-Nguanmoo et al. 2017). Ibrahim et al. reported that dapagliflozin improved spatial memory, and mitigated AD-associated histopathological changes in the ovariectomized/D-galactose rat model of AD (Ibrahim et al. 2022). Apaijai et al. investigated whether dapagliflozin can improve cognitive function and synaptic plasticity in rats with myocardial infarction (MI) in comparison with angiotensin-converting enzyme inhibitor. Dapagliflozin enhanced glutamate levels in the brain. Nevertheless, it was insufficient to improve cognitive impairment, synaptic dysplasticity, and dendritic spine loss in MI rats (Apaijai et al. 2025). Hierro-Bujalance et al. analyzed the role of empagliflozin at the central level in an AD-T2D mouse model. Empagliflozin limited cortical thinning, reduced neuronal loss, and ameliorated cognitive deficits (Hierro-Bujalance et al. 2020). Davri et al. examined whether empagliflozin could be a viable candidate for managing AD. Empagliflozin preserved dendritic spine density. In addition, a modest improvement in dendrite length was observed (Davri et al. 2024).

SGLT2 inhibitors can cross the blood-brain barrier. They reduce neuroinflammation and oxidative stress in the hippocampus and may have neuroprotective effects such as reducing the amyloid burden and inhibiting AChE (Kostrzewska et al. 2025; Stanciu et al. 2023). Arafa et al. studied the effect of canagliflozin as compared to galantamine for 2 weeks in rats with scopolamine hydrobromide-induced memory dysfunction. Researchers conclude that orally administered canagliflozin might improve memory dysfunction via cholinergic and monoaminergic systems (Arafa et al. 2017). Stanciu et al. analyzed the effect of canagliflozin and donepezil for 21 days on AD-relevant behaviors and brain pathology in mice. As a result of the study, canagliflozin (using gavage) improved the behavioral parameters in the novel object recognition test and the elevated plus maze. Moreover, it decreased AChE activity (Stanciu et al. 2023). Khamies et al. studied with STZ-induced sporadic AD mice model. Canagliflozin administered orally for 21 days restored cognitive deficits (Khamies et al. 2024). Elariny et al. elucidated the potential effects of different doses of canagliflozin on AD induced by aluminium chloride in rats. Canagliflozin administered orally for 35 days elicited significant dose-dependent changes in the Morris Water Maze test and object recognition test (Elariny et al. 2024). In our study, canagliflozin was i.c.v. administered for 7 days, so it is possible that canagliflozin did not affect behavioral characteristics.

Jayarathne et al. show that canagliflozin improved hippocampal-dependent learning and memory in males but failed to rescue cognition in females. Furthermore, it decreased the burden of amyloid plaque in males only (Jayarathne et al. 2025). Based on this study, the inclusion of different gender groups in further studies can reveal the differences between genders in the observed dendritic changes.

This study focuses on the morphological effect of canagliflozin on the AD model. Our limitations include the fact that the AD model was validated only with behavioral parameters and that there was no naive control group in the morphological analysis.

## Conclusion

In summary, there are significant alterations in the dendrite morphology of pyramidal neurons in the hippocampus of AD model treated with canagliflozin and donepezil. In the CA1 region, canagliflozin was as effective as donepezil on dendrite parameters. However, canagliflozin was not as effective as donepezil in improving memory-related parameters of behavioral tests.

The current study detected the neuroplastic effect of canagliflozin. This morphological framework, indicating dendritic plasticity and remodeling, serve to better understand the cellular effects of canagliflozin. Therefore, our study may contribute to the development of novel strategies for therapy of AD. In further studies, combination therapy may be used to evaluate the potential to slow the progression of AD pathology.

## Data Availability

The datasets generated during and/or analyzed during the current study are not publicly available. However, we will be a pleasure to share the data once the publication is accepted.
